# Accurate Diagnosis of Small Ruminant Lentivirus Infection Is Needed for Selection of Resistant Sheep through TMEM154 E35K Genotyping

**DOI:** 10.3390/pathogens10010083

**Published:** 2021-01-19

**Authors:** Hugo Ramírez, Irache Echeverría, Alfredo A. Benito, Idoia Glaria, Julio Benavides, Valentín Pérez, Damián de Andrés, Ramsés Reina

**Affiliations:** 1 Virology, Genetics and Molecular Biology Laboratory, Faculty of Higher Education, Cuautitlan, Veterinary Medicine, Campus 4, National Autonomous University of Mexico, Km. 2.5 Carretera Cuautitlán-Teoloyucan, San Sebastián Xhala, Cuautitlán Izcalli Estado de México C.P. 54714, Mexico; ramiralh@unam.mx; 2 Animal Health Department, Institute of Agrobiotechnology (IdAB), CSIC-Government of Navarra, 31192 Navarra, Spain; irache.echeverria@unavarra.es (I.E.); idoia.g@csic.es (I.G.); damian.deandres@csic.es (D.d.A.); 3 Molecular and Cell Biology Department, EXOPOL SL, 50840 Zaragoza, Spain; abenito@exopol.com; 4 Mountain Livestock Institute (IGM), CSIC-University of León, 24346 León, Spain; julio.benavides@csic.es; 5 Department of Animal Health, University of León, 24071 León, Spain; valentin.perez@unileon.es

**Keywords:** small ruminant lentiviruses, TMEM154, ELISA, PCR

## Abstract

Small ruminant lentiviruses (SRLV) cause an incurable multiorganic disease widely spread in sheep and goats that disturbs animal welfare and production. In the absence of a vaccine, control measures have been traditionally based on early diagnosis and breeding with virus-inactivated colostrum with segregation of seropositive animals. However, antigenic heterogeneity, poor antibody production due to low viral load, and single strain design of most available ELISA, pose a threat to SRLV diagnosis. Genome-wide association studies have described TMEM154 E35K polymorphism as a good genetic marker for selection of resistant animals in some American and European breeds. In this study, a multitargeted serological and virological screening of more than 500 animals from four different breeds (latxa, raza Navarra, assaf, and churra) attending to SRLV infection status was performed. Then, animals were genotyped to characterize TMEM154 E35K polymorphism. ELISA procedures, individually considered, only identified a proportion of the seropositive animals, and PCR detected a fraction of seronegative animals, globally offering different animal classifications according to SRLV infection status. TMEM154 allele frequency differed substantially among breeds and a positive association between seroprevalence and TMEM154 genotype was found only in one breed. Selection based on TMEM154 may be suitable for specific ovine breeds or SRLV strains, however generalization to the whole SRLV genetic spectrum, ovine breeds, or epidemiological situation may need further validation.

## 1. Introduction

Small ruminant lentivirus (SRLV) infection widely affects animal health and production by causing a wasting disease characterized by chronic inflammation of carpal joints, udder, central nervous system, and/or lungs [1]. Infection takes place through colostrum/milk consumption from infected ewes, and/or by direct contact with respiratory secretions from infected animals [2,3]. Once infection occurs, immune responses result in production of antibodies that generally remain detectable, for the whole animal’s life, which is at the basis of the current control programs [4,5]. Strategies to control SRLV are based on the identification of seropositive animals since no vaccine is available, despite profuse trials [6]. However, antigenic heterogeneity of circulating strains may be wider than the covered by available ELISA tests [7,8,9] making serological response not always detectable. Accordingly, the description of new infection outbreaks in ELISA-controlled flocks [9,10,11] have practically questioned current control strategies. Molecular diagnosis by PCR may add diagnostic value to serodiagnosis since seronegative animals may show PCR positive results due to low antibody production [12,13]. New molecular methods are being described focused on the design of universal primers, thereby increasing sensitivity to enable the identification and removal of animals with low viral load in vivo [14,15,16,17].

In addition to their use in control programs, the detection of antibodies through ELISA tests along with molecular tools has been used to identify association between breeds and susceptibility patterns to SRLV infection. Genome wide association studies (GWAS) have opened the possibility to apply genetic selection programs by describing a number of candidate genes associated to SRLV seroreaction [18,19,20,21] or proviral load [22]. Among them, different studies suggest that TMEM154 haplotypes 1, 2, and 3, the most common haplotypes found in sheep, have an effect on SRLV susceptibility. Sheep with a copy of either haplotype 2 or 3, both of which encode a glutamate amino acid residue at position 35 (E35) of the extracellular portion of TMEM154, have an increased risk of SRLV infection. Conversely, sheep homozygous for haplotype 1, which encodes a lysine residue at position 35 (K35), have a decreased risk of infection in sheep breeds from North America and Germany [18,23,24], but also in Asian sheep breeds [25,26]. With the exception of the mentioned countries, there is little information about the TMEM154 haplotype composition in productive breeds and its association with SRLV susceptibility.

In this study, we analyzed TMEM154 E/K genotype association with SRLV infectious status in approximately 500 sheep belonging to different flocks, production systems, and breeds according to three different ELISAs and a PCR-based molecular test.

## 2. Results

### 2.1. Serodiagnosis

SRLV diagnosis through ELISA was carried out using three different commercial tests based on different strains and antigens. An animal was considered infected in the Total ELISA classification when tested positive to at least one of the ELISAs.

At the population level, ELISA testing indicated that all the flocks were infected with a seroprevalence ranging from 3.5% to 100%. Individually considered, the different ELISAs showed important differences when examining intraflock seroprevalence. Interestingly, two flocks of the churra breed were classified as uninfected taking into account results from ELISA#1. However, Total ELISA analysis indicated a seroprevalence of 60% (Table S1).

Considering animals by breed, assaf flocks were the most infected showing a seroprevalence up to 89%, depending on the ELISA tested. When considering the results from the three ELISA tests, seropositive animals reached 97.3% (Table 1), being all flocks above 90% of seroprevalence. Raza and latxa Navarra breeds showed a moderate seroprevalence according to single ELISA tests, however, when applying the three ELISAs seropositive animals reached 50%. Churra sheep showed moderate to high seroprevalence values, reaching a total ELISA rate of 66.3% (Table 1). ELISA efficiencies, calculated as the proportion of seropositive animals detected by a single ELISA, reached 56%, 74%, 92%, and 91% in raza Navarra, latxa Navarra, assaf, and churra animals, respectively. ELISA#3 was clearly more performant in churra animals, whereas infection in latxa Navarra animals was better detected by ELISA#1 (Table 1).

Interestingly, the combination of all ELISAs revealed a global seroprevalence higher than 65%, practically doubling the performance offered by kits individually considered. Indeed, efficiency of individual ELISAs varied from 0% to 100% according to flocks (Table S1) and from 38.46% to 91.67% depending on the breed (Table 1).

### 2.2. Molecular Diagnosis

Diagnosis through commercial PCR resulted as sensitive as ELISA, since the overall PCR reactivity was around 44% compared to 46.4%, 44.9%, and 42.7% for ELISAs #1, #2, and #3, respectively (Table 2). As shown for serological analysis, PCR reactivity also depended on the flock considered, since 77% of the animals were detected in assaf flocks and only 22% in the churra animals (Table S2).

**Figure 1:**
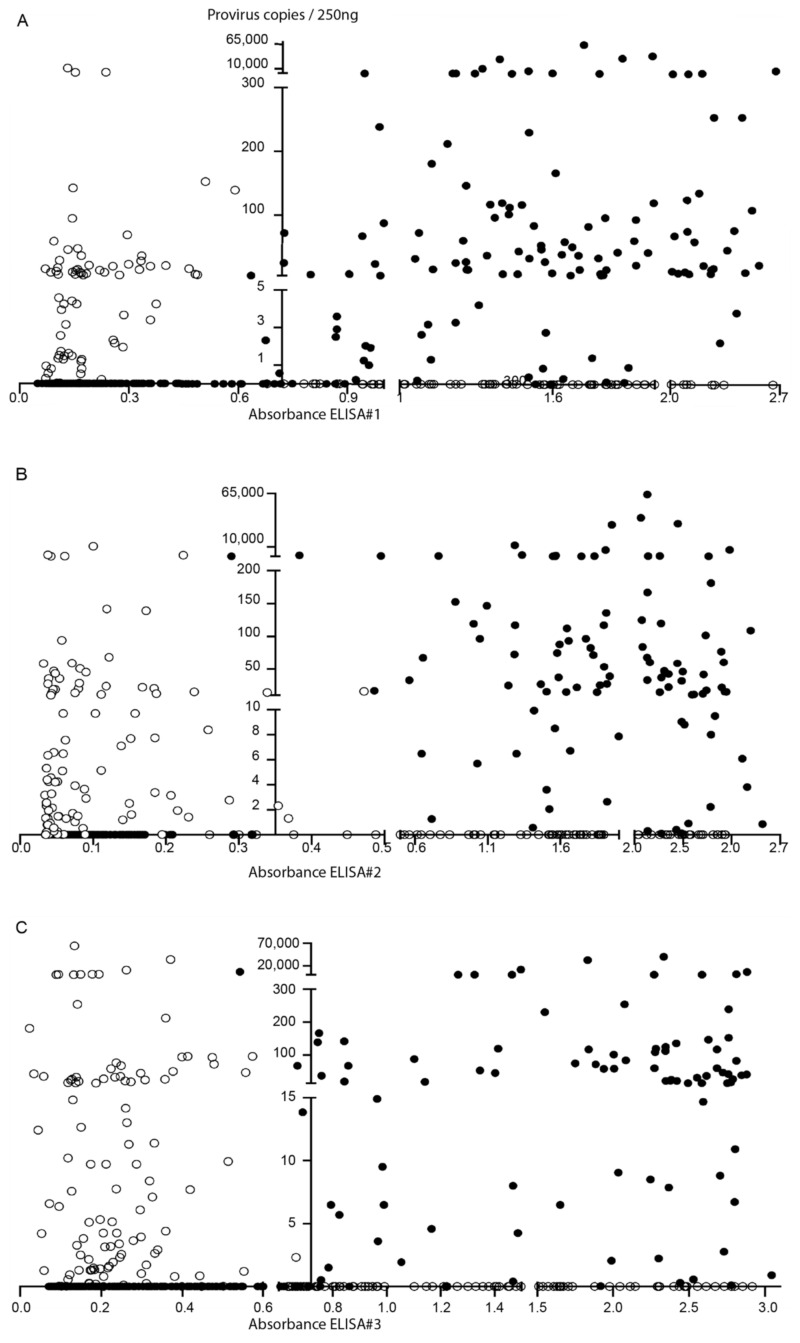
Small ruminant lentivirus (SRLV) diagnosis. Scatter plot distribution of ELISA absorbance (X-axis) and proviral load in 250 ng of DNA (Y-axis) data. Samples concordantly positive or negative between ELISA#1 (**A**), ELISA #2 (**B**), or ELISA#3 (**C**) and PCR (●) and discordant samples (O) are represented. The Y-axis intercepted the X-axis at the average value of the corresponding ELISA positivity threshold.

Among seronegative samples, 54 out of 179 (30%) were identified as qPCR positive, whereas 135 seropositive samples resulted negative in qPCR. When considering each ELISA individually, PCR detected a 22.37%, 34.53%, and 37.94% of seronegative animals to ELISAs #1, #2, and #3, respectively (Figure 1 and Tables S4–S6).

Total infected animal classification, revealed by ELISA or PCR, allowed the evaluation of PCR efficiency compared to ELISA. qPCR efficiency reached 79% in assaf animals and decreased to 32% in churra flocks. Intraflock efficiency in assaf flocks peaked at 95.8%, whereas highly seropositive churra flocks were not detected by qPCR (Table S2).

Animal classification into infected and uninfected after ELISA (Table S3) and qPCR proviral quantification is represented in Figure 1.

### 2.3. TMEM154 Genotyping

**Figure 2:**
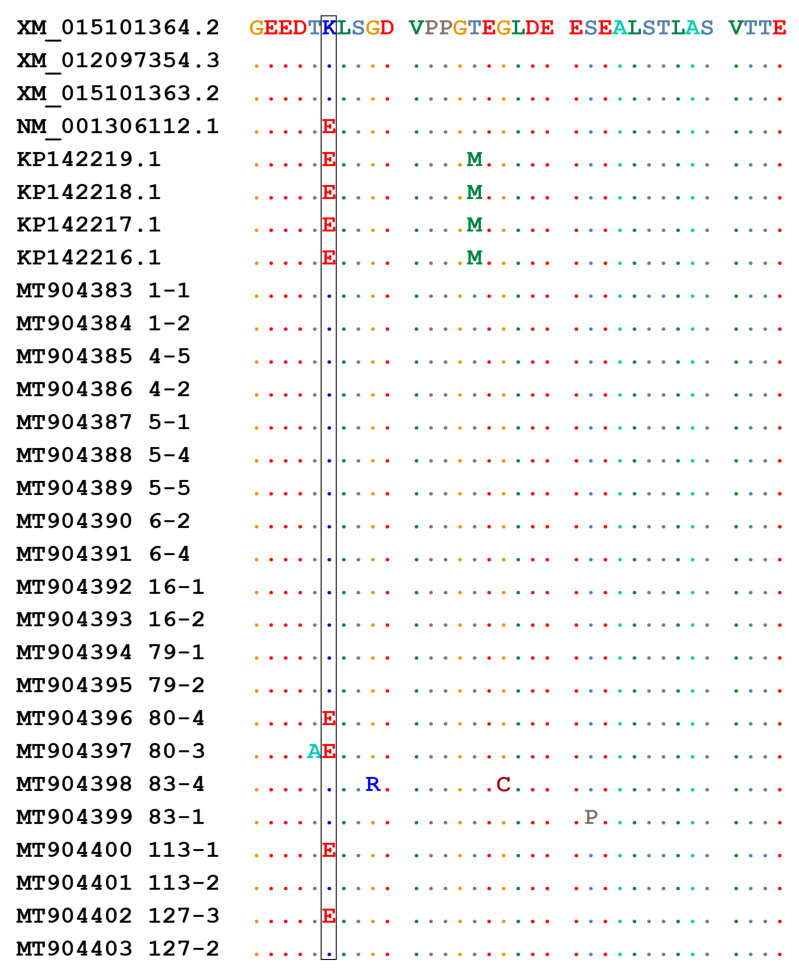
Identification of TMEM154 E35K genotype. Alignment of partial TMEM154 sequences obtained from selected sheep. Numbers refer to the animal sample and clone analyzed. Amino acid substitution at position 35 is highlighted. Identical residues are indicated by dots.

Ovine DNA samples (*n* = 10) from the studied population were employed to amplify a 335bp region of the TMEM154 gene (Table 3), including residue at position 35, that was cloned and sequenced (Figure 2).

Considering Sanger sequencing, seven samples were identified as homozygotes for allele 1, one as homozygote for allele 2, and two as heterozygotes. Specific clones encoding allele 1 or allele 2 were used for real time PCR standardization.

**Figure 3:**
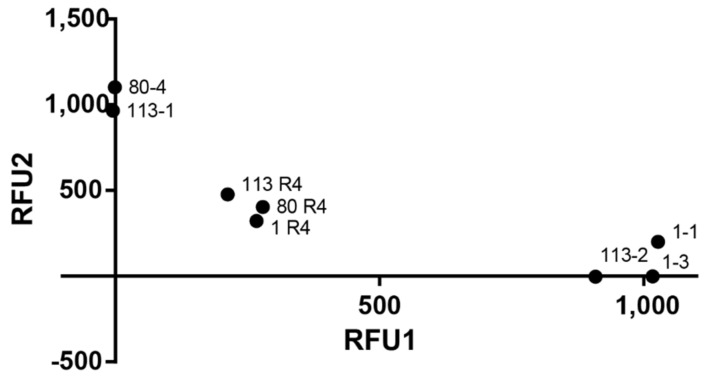
TMEM E35K genotyping using fluorogenic probes. Scatter plot distribution of relative fluorescence of FAM, representative of allele 1 (X-axis) and HEX (allele 2; Y-axis) of TMEM154 clones. Original animal samples are also shown showing a heterozygote pattern.

Fluorogenic probes were designed within the E35K SNP, with either FAM or HEX (Table 3), to specifically detect plasmids encoding the corresponding genotype. Equimolar mixes of plasmids encoding each of the alleles were automatically classified as heterozygotes, validating their application in biological samples (Figure 3).

Allelic discrimination analysis showed different allele frequencies according to the breed considered (Table 4). The protective genotype (K/K) was predominant in all breeds analyzed, followed by heterozygotes and homozygotes (E/E), except for the assaf breed in which heterozygotes and homozygotes (E/E) were prevalent.

### 2.4. TMEM154 E35K Association with SRLV Infection Status

**Figure 4:**
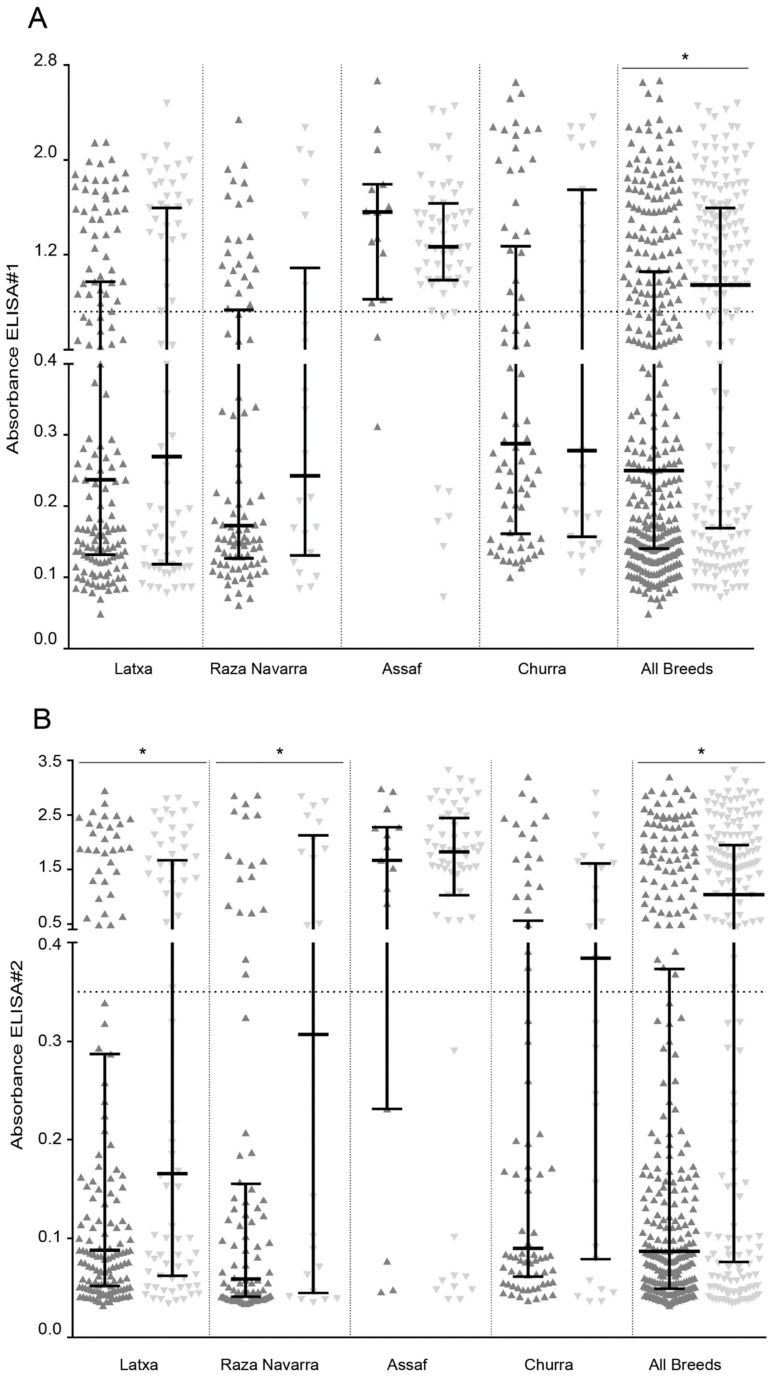

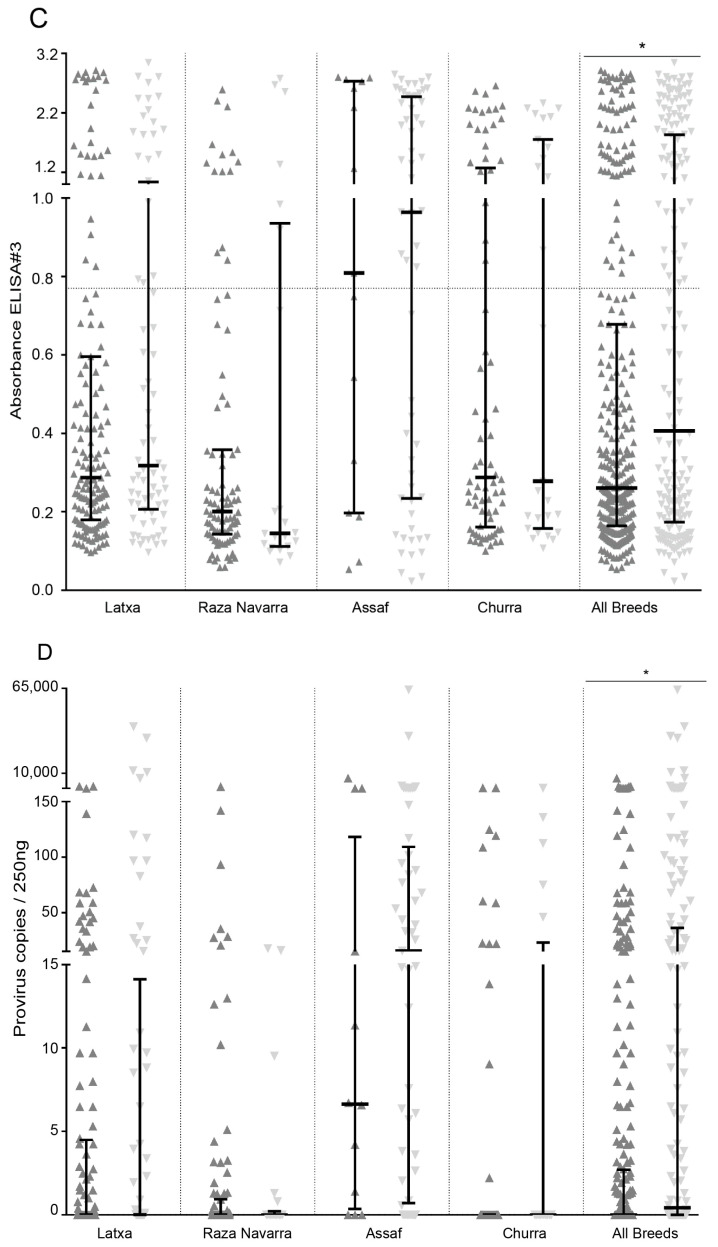
Distribution of ELISA#1 (**A**), ELISA#2 (**B**), ELISA#3 (**C**) absorbance and qPCR proviral load (**D**) according to TMEM154 genotyped latxa, raza Navarra, assaf, and churra sheep. Animal samples were classified according to the E35K TMEM154 polymorphism into K/K (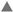) or E/K and E/E (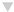) and analyzed by ELISA and qPCR. Samples were grouped by individual breeds and combined (All breeds). Average cut-off values of individual ELISA are represented as a horizontal dotted line (* Mann–Whitney, *p* < 0.05).

Genotyped sheep were distributed according to ELISA absorbance and PCR proviral load (Figure 4). Considering breeds in which the K/K allele was predominant, the proportion of seropositive and seronegative samples in ELISAs #1 and #3 was similar in resistant (K/K) or susceptible (E/K and E/E) genotypes (Table 5). Similarly, assaf animals were mostly seropositive irrespective of their TMEM154 genotype (Tables S4–S6). However, when considering all breeds as a whole, significant difference was found between resistant and susceptible genotyped samples, mean absorbance being higher in susceptible samples (*p* < 0.05 Mann–Whitney). Exceptions to this general picture were evident when analyzing data obtained after ELISA#2 testing of latxa and raza Navarra breeds, since differences were found in ELISA absorbance according to TMEM154 genotype (Figure 4B and Table S5).

Distribution of genotyped samples according to proviral load values was similar among resistant (K/K) and susceptible (E/K and E/E) samples, suggesting poor association between TMEM154 genotype and SRLV infection (Figure 4D).

Relationship between SRLV infection status and TMEM154 genotyping was evaluated using association and relative risk, and regression statistical analyses. Animals from the assaf and churra breeds did not show significant association between TMEM154 genotype and SRLV antibody occurrence, except for ELISA#2 in churra animals (Table 5). Similarly, animal classification by ELISA#2 of the raza Navarra and latxa Navarra breeds allowed a significant association between SRLV seroreactivity and TMEM154 genotype. Additionally, reactivity to ELISAs #1, 2, and 3 was also associated to TMEM154 genotype in latxa Navarra animals. Total ELISA reactivity was associated to genotyping in the case of the aforementioned breeds (raza Navarra and latxa Navarra), but not in assaf or churra sheep.

Molecular diagnosis led to a classification of animals into infected or uninfected that was not associated with TMEM154 genotype in the sheep analyzed. However, when combining PCR and ELISA results (Total infected), association was found in the case of the latxa Navarra breed. The relative risk of being seropositive when encoding a susceptible genotype, homozygous or heterozygotes, was moderate within quoted significant associations, and varied from 1.54 to 3.59 (Table 5). Identical results were obtained when applying a generalized linear model of association.

## 3. Discussion

Lentivirus infection remains one of the major threats in ovine and caprine species in spite of the surveillance and control programs driven from the 1990s in different countries by serological screening with available tools. However, ELISA testing has some inherent disadvantages that jeopardize SRLV diagnosis. On one hand, selection of diagnosis escape mutants could explain previously described diagnostic concerns [9,11,27]. On the other hand, the antigenic spectrum of SRLV, constantly enlarged by descriptions of new genotypes and subtypes [28], is not fully covered by commercial ELISA [9,29,30,31], at least when applied individually [7]. Both factors account for the variable proportion of infected animals not recognized by available ELISAs, as revealed by molecular methods. In the search for alternative tools in the design of control measures, genetic selection through TMEM154 genotyping has been proposed in the SRLV field [18]. TMEM154 genotyping based on E35K position has been associated to SRLV infection in different American, European, and Asian ovine breeds and stands as the most promising candidate so far [24,25,26]. This study aimed at uncovering the potential use of TMEM154 genetic selection in sheep belonging to different breeds and production systems, and infected with different SRLV genotypes [7]. The first approach was to unequivocally identify SRLV infected animals through application of a multiplatform strategy including serological and molecular strategies. Stratified data allowed the analysis of association between TMEM154 genotype and SRLV infection status.

Serological screening revealed that antibody detection using more than one ELISA test significantly improved diagnosis, since the proportion of positive animals considerably increased when the three tests were included. Individual ELISA efficiency in seropositive samples varied among flocks being higher in heavily infected flocks, whereas in those showing moderate seroprevalence, efficiency decreased to 0%. Increased seroprevalence recorded in assaf flocks may be due to the intensive dairy production system that implies close contact between animals and long-term indoor housing, contributing to increased virus transmission [32]. Interestingly, two churra flocks would have been diagnosed as uninfected when using ELISA#1, or underestimated using ELISA#2. Despite these data, none of the ELISA could be chosen as the best option to detect SRLV infected animals according to the tested population, since ELISA performance varied considerably depending on the flock considered. Animal management system or breed can be excluded from the possible reasons since differences in ELISA performance between similar managed flocks composed of different breeds were evident, as it occurred with semi-intensive churra, latxa, and raza Navarra or in intensive assaf breeds.

One possible explanation may rely on the unmatched antigenic ELISA design with the circulating strains. Sheep are likely infected by a mix of lentiviruses including strains of different genotypes, even within the same flock or individual [33,34,35], thereby enlarging the antigenic repertoire to be detected. Cross-reaction paradigm among Maedi Visna (genotypes A1–3) and CAEV (genotype B1) strains was described in the 1990s when only few genotypes were known [36,37,38,39]. However, molecular methods have allowed the discovery of more than 25 novel subtypes within genotypes A [40] and B [28], and even completely new genotypes such as C [41] and E [42] in recent years. Indeed, a variable proportion of seronegative samples have been evidenced as infected by PCR in this study and elsewhere [13,17,43,44]. Accordingly, efficiencies of the individual tests, regardless serological or molecular, when referred to the Total infected result were low. In this situation, an adequate strategy, involving multiple ELISA testing combined with molecular methods, should be ideally established, not only for epidemiological or control purposes, but also in studies evaluating two cohorts of infected vs uninfected animals when assessing genetic resistance or production losses. Accurate identification of infected animals will reduce the risk of perpetuating the infection in controlled flocks. Additionally, new diagnostic strategies, based on both ELISA and PCR, should be updated aiming at detecting animals infected with new SRLV antigenic variants.

Alternative control strategies explored so far include genetic selection of resistant variants. Based on genome-wide association studies (GWAS), TMEM154 specific alleles at position 35 (E/K) have been associated to SRLV infection susceptibility, in terms of serological reaction [18,24,26], but also proviral load [20]. Up to date, susceptibility to SRLV in animals showing homozygous (E/E) or heterozygous (E/K) genotypes has been tested in American, but also European and Asian ovine breeds. Since a natural susceptibility may account for the differences found in ELISA and qPCR performances presented in this work, a TMEM154 genotyping method was developed and applied to all the animals.

Fluorogenic probes were efficient and easy-to-design tools to genotype ovine DNA for E35K SNP. Allele frequency reflected previous observations and did significantly differ among breeds. Raza Navarra, a meat-oriented breed, showed a KK-resistant genotype frequency higher than 80%, very similar to previous results obtained in rasa Aragonesa, a closely related breed [19]. Despite the TMEM154 resistant profile found, SRLV infection is widely distributed in this breed (this study and [4], as it is in the milk-oriented breed latxa Navarra, which also showed a high frequency of the resistant genotype [18,45]). Churra flocks despite showing a prevalent frequency of the resistant genotype, were all seropositive at different degrees depending on the diagnostic test applied. By contrast, assaf sheep showed a high frequency of the susceptible genotype, either homo or heterozygote, of around 80% that, however, was not associated with SRLV infection. Whether this is extensive to other productive breeds showing resistant genotypes, as lacaune for example (Table S4), is currently unknown.

Among breeds studied, only latxa breed showed a constant association between TMEM154 genotyping and SRLV infection status regardless of the test used for animal classification. Similarly, raza Navarra animals classified by ELISA#2 as uninfected more likely encoded a K/K resistant genotype. Regarding proviral load, no association was found with TMEM154 genotype as shown in Figure 4D. Meaningful association with proviral load should be evaluated not only in the context of infection status, but also involving evaluation of clinical signs in studied animals.

The lack of association described in this study may be due to the existence of other missense mutations within TMEM154 gene different from E35K such as D33N, T44M, I70N, or G38R [26] that may link TMEM154 with SRLV susceptibility in these breeds. The potential involvement of these SNPs individually or jointly considered in SRLV susceptibility is unknown. Additional genotyping of these samples may uncover this possibility. In addition, high infection pressure present in flocks analyzed (most of them were above 50% of infected animals) may have overwhelmed association with TMEM154. However, similar infection pressure has been observed in German flocks, in which establishment of statistical association was possible [24].

Another explanation may rely on the SRLV circulating strains and not related to the host. This association was firstly described in animals from the United States and infected with a genotype A2 strain, that may not require a functional TMEM154 to infect sheep [46]; Turkish sheep included in the same study are likely infected by an ancestral genotype A variant [47]; SRLVs infecting German and Iranian sheep have not been characterized so far [25]. Circulating strains in the flocks included in this study are likely a mix of lentiviruses belonging to genotypes A and B, taking into account differential ELISA reactivity, and partial genetic characterization (data not shown). The main difference with the aforementioned studies was the diagnostic strategy used that implied multiple ELISA testing combined with molecular detection of provirus.

Little is known about TMEM154 function; apart from the transmembrane location, GWAS studies have identified TMEM154 as a candidate for asthma severity [48] and for type-2 diabetes in a meta-analysis, which combined GWAS data from multiple human ethnic groups, including European, East Asian, South Asian, and Mexican/Mexican American [49,50,51,52], both studies relating TMEM154 to inflammatory processes. Disease caused by SRLV is characterized by inflammation, potentially relating TMEM154 expression with development of inflammation in target tissues.

Our results suggest that the relationship between TMEM154 E/K genotyping and susceptibility patterns when facing SRLV infection is not clear for all breeds and SRLV genotypes, and should be tested in a case-by-case manner in order to avoid selection of infected animals as resistant. Combined serological and molecular diagnosis are highly recommended to accurately classify infected animals in order to provide robust studies.

## 4. Materials and Methods

### 4.1. Animals and Samples

Blood samples of the latxa Navarra (two flocks, *n* = 194), raza Navarra (two flocks, *n* = 114), assaf (four flocks, *n* = 74), and churra (10 flocks, *n* = 101) ovine breeds from northern Spanish flocks were obtained. All sheep belonged to 18 different flocks dedicated to dairy or meat production. Flocks 1 and 2 from the raza Navarra breed (meat flocks focused on semi-intensive lamb production), and flocks 4 and 5 from the Latxa Navarra breed (dairy flocks, focused on semi-intensive milk production combining free grazing periods with housing) were likely infected by different genotypes of SRLV [28]. Assaf and churra sheep were from intensive and semi-intensive dairy farms, respectively, located in Castilla y León, except flock 3 from assaf breed that was located in Navarra. None of the studied animals presented clinical signs of SRLV disease.

Whole blood was obtained in EDTA-K3+ tubes by jugular puncture. After centrifugation, plasma samples were stored at −20 °C until use in ELISA. Buffy coats were washed, erythrocytes lysed, resuspended in PBS, and stored at −20 °C until DNA extraction.

### 4.2. Serological Survey

Plasma samples were tested for the presence of SRLV antibodies with three commercial ELISA kits: EradikitTM SRLV screening test (In3 Diagnostic, Torino, Italia, ELISA#1) [31]; ELITESTTM MVV/CAEV (Hyphen Biomed, Neuville-sur-Oise, France, ELISA#2) [39] and INgezim Maedi screeningTM (Ingenasa, Eurofins Technologies, Madrid, Spain, ELISA#3) [30]. All tests were performed following manufacturers’ instructions. Data were analyzed by considering each ELISA individually and combined. Samples positive to at least one of the ELISA tested were considered in “Total ELISA” results.

### 4.3. DNA Extraction and Quantification

Genomic DNA was extracted from buffy coat samples with E.Z.N.A. tissue/blood kit (OMEGA, Bio-tek, Norcross, GA, USA) following the manufacturer’s instructions. DNA was quantified at 260–280nm (Nanodrop Onec, Thermo Scientific®, Waltham, MA, USA) and stored at −20 °C until use.

### 4.4. SRLV Molecular Diagnosis

Real time PCR was performed with 250 ng of DNA in an Agilent sequence detector system using the commercial kit EXOone Maedi Visna-CAEV oneMix kit, following manufacturer’s instructions (Exopol, Zaragoza, Spain). Six-fold serial dilutions of the positive control were prepared to generate a standard curve (cycle threshold vs. copy number) from which copy number values were extrapolated. Positive control copy number ranged from 5 × 10^5^ to 5. Results were expressed as provirus copy number/250 ng of DNA.

An animal was considered as infected when at least one ELISA test or one PCR method revealed a positive result (Total Infected).

### 4.5. TMEM154 Genotyping

Setting-up TMEM154 E/K genotyping involved a first step, in which 10 DNA samples from SRLV seronegative (5) and seropositive (5) latxa Navarra animals were used. Amplification of the corresponding TMEM154 region (Table 2) following standard PCR procedures, cloning in pGEMT-easy plasmids (Promega, Madison, WI, USA) and sequencing (STAbVida, Caparica, Portugal), were carried out. Based on the obtained sequences, specific primers and fluorogenic probes were designed using Primer Express® Software (Applied Biosystems, San Francisco, CA, USA). Each probe was specifically designed to match the E35K mutation, E version was synthesized with HEX and K version with FAM. Both reporters were quenched with BHQ-1 (Table 1). Real time PCR was carried out in Buffer 1x (Biotools, Madrid, Spain), 1.5 MgCl_2_ (Biotools, Madrid, Spain), 230 μM dNTPs (Applied Biosystems, Warrington, UK), 400 nM of forward and reverse primer, 200 nM of each probe (Metabion, Planegg, Germany), 0.04 U/uL of Taq DNA polymerase (Biotools, Madrid, Spain) in a final volume of 25 μL. Samples were submitted to an initial denaturation at 95 °C/5 min, followed by 45 cycles of 55 °C 30 s. Allelic discrimination was analyzed using BIO-RAD CFX96 software. This new method for ovine TMEM154 genotyping was evaluated with plasmids encoding each of the versions (E or K) obtained from the sequencing. Equimolar mixes of these plasmids were mixed to mimic heterozygote samples. Plasmid controls encoding E, K alleles or the equimolar mix were included in each plate when analyzing biological samples.

### 4.6. Statistical Analysis

Diagnostic efficiency was determined for each of the ELISAs in comparison with the total seropositive (Total ELISA) and infected (Total infected) population. Efficiency of diagnostic PCR was calculated as regarding to the Total infected.

Differences in the distribution of allele frequencies between groups of SRLV infected and uninfected samples were tested by Fisher´s exact test. The relative risk (RR) to be detected by ELISA or PCR was estimated for animals carrying one and/or two copies of the putative susceptible allele (risk factor) with the method of Altman [38]. Nonparametric Mann–Whitney tests were used to compare ELISA absorbance and proviral load values among TMEM154 genotyped groups. Association between TMEM154 genotype and SRLV infection status was performed using regression through generalized linear model.

The SPSS program (v. 25.0) for Windows was used for statistical analyses and alpha error was set at 0.05.

## 5. Conclusions

The combination of different serological and molecular methods was useful and needed to accurately classify animals into SRLV infected or uninfected. Combined diagnosis significantly improved performance of tests individually considered. TMEM154 frequencies of raza Navarra, latxa Navarra, and churra breeds resembled those of resistant sheep, however, infection rate was high as determined by the combined strategy used. SRLV infection status was associated with TMEM154 genotyping only in latxa navarra animals.

Antigenic heterogeneity of SRLV greatly challenges accurate serological diagnosis with available methods, and commercial molecular tests are currently passing from the bench to the market demonstrating a convincing benefit. It is not only SRLV control or surveillance programs that are profoundly affected by this diagnostic drawback, but also scientific studies requiring strict animal classification into infected and uninfected animals. Controversy in studies evaluating production losses derived from SRLV infection, or linking host genetic features with specific traits, may have suffered from this inaccuracy when identifying infected animals.

TMEM154 involvement on SRLV patterns of susceptibility may require a further evaluation in specific breeds and genotypes of SRLV.

## Figures and Tables

**Table 1:** Small Ruminant Lentivirus (SRLV) seroprevalence and ELISA efficiency in raza Navarra, latxa Navarra, assaf, and churra ovine breeds. Total ELISA reflects reactivity to any of the ELISAs used.

TEST	Raza Navarra				Latxa Navarra				Assaf				Churra			
	*n*	Positive		Efficiency %	*n*	Positive		Efficiency %	*n*	Positive		Efficiency %	*n*	Positive		Efficiency %
			*n*			%	*n*			%	*n*			%	*n*		%
ELISA#1	114	29	25.4	55.8	194	76	39.2	73.8	74	66	89.2	91.7	101	32	31.7	47.8
ELISA#2	114	28	24.6	53.9	194	60	30.9	58.3	74	61	82.4	84.7	101	42	41.6	62.7
ELISA#3	114	20	17.5	38.5	194	60	30.9	58.3	74	46	62.2	63.9	101	61	60.4	91.0
Total ELISA	114	52	45.6	100.0	194	103	53.1	100.0	74	72	97.3	100.0	101	67	66.3	100.0

**Table 2:** Small Ruminant Lentivirus (SRLV) provirus detection using real time quantitative PCR (qPCR). Total infected refers to samples positive to any of the diagnostic methods used (ELISA and/or PCR).

TEST	Raza Navarra				Latxa				Assaf				Churra			
	*n*	Positive		Efficiency %	*n*	Positive		Efficiency %	*n*	Positive		Efficiency %	*n*	Positive		Efficiency %
			*n*			%	*n*			%	*n*			%	*n*		%
qPCR	111	33	29.7	45.2	191	90	47.1	65.7	74	57	77.0	79.2	82	18	22.0	32.1
Total infected	114	75	65.8	100.0	194	139	71.6	100.0	74	72	97.3	100.0	101	67	66.3	100.0

**Table 3:** Primer and probe sequences, amplification product size, and purpose of the corresponding PCR method.

Probes/Primers Sequences	Product Size (Base Pairs)	Purpose
Fw 5′-CTGCCTTTGTGGGAGATTTA-3′	335	Amplification and sequencing for verification of genotyping results
Rv 5′-TTCTGTGGTCACTGAAGCAA-3′		
Fw 5′-TTCGTCTCCATGACAAGTCTCAAT-3′	121	Determination of nucleotide substitution G/A, resulting in amino acid substitution E35K.
Rv 5′-GCTTAGGGCCTCTGACTCTTCA-3′		
HEX-AGGACACAGAACTGT-BHQ-1		
6-FAM-AGGACACAAAACTGT-BHQ-1		

**Table 4:** Allelic frequency among TMEM154 E35K genotyping in raza Navarra, latxa, assaf, and churra ovine breeds.

Genotype	Raza Navarra		Latxa		Assaf		Churra	
	*n*	%	*n*	%	*n*	%	*n*	%
K/K	92	80.7	134	69.1	15	20.3	75	74.3
E/K	18	15.8	56	28.9	32	43.2	24	23.8
E/E	4	3.5	4	2.1	27	36.5	2	2.0
Total	114	100	194	100	74	100	101	100

**Table 5:** Small Ruminant Lentivirus (SRLV) infection status and TMEM154 genotyping association. Samples classified into positive or negative according to different methods (ELISAs and qPCR) were re-classified according TMEM154 E35K polymorphism. Statistical probability associated to Fisher’s exact test (p) and to relative risk (RR; p’) are shown. Significant values are in bold.

TEST	SRLV	Raza Navarra						Latxa Navarra					Assaf					Churra			
		TMEM154 Genotype (%)					TMEM154 Genotype (%)					TMEM154 Genotype (%)					TMEM154 Genotype (%)				
				*p*	RR	*P’*			*p*	RR	*P’*			*p*	RR	*P’*			*p*	RR	*P’*
		KK	EK/EE		(95%CI)		KK	EK/EE		(95%CI)		KK	EK/EE		(95%CI)		KK	EK/EE		(95%CI)	
ELISA#1	Negative	69	16	0.792	1.09 (0.51–2.35)	0.824	95	23	**<0.0001**	2.12 (1.52–2.95)	**<0.0001**	2	6	0.66	1.04 (0.83–1.29)	0.745	53	16	0.338	1.35 (0.74–2.48)	0.326
	Positive	23	6				39	37				13	53				21	10			
ELISA#2	Negative	74	12	**0.024**	2.32 (1.25–4.31)	**0.008**	111	23	**<0.0001**	3.59 (2.36–5.48)	**<0.0001**	4	9	0.446	1.16 (0.84–1.60)	0.381	50	9	**0.005**	2.02 (1.31–3.10)	**0.002**
	Positive	18	10				23	37				11	50				24	17			
ELISA#3	Negative	78	16	0.214	1.79 (0.78–4.13)	0.171	101	33	**0.007**	1.83 (1.22–2.75)	**0.0037**	5	23	0.772	0.91 (0.60–1.38)	0.674	31	9	0.644	1.11 (0.79–1.56)	0.530
	Positive	14	6				33	27				10	36				44	17			
TOTALELISA	Negative	52	10	0.475	1.25 (0.80–1.96)	**0.003**	73	18	**0.002**	1.54 (1.20–1.98)	**0.0007**	0	2	1	0.97 (0.92–1.01)	0.157	28	6	0.232	1.23 (0.93–1.61)	0.142
	Positive	40	12				61	42				15	57				47	20			
qPCR	Negative	62	16	1	0.90 (0.42–1.91)	0.781	72	29	0.437	1.15 (0.84–1.56)	0.384	3	14	1	0.95 (0.71–1.27)	0.747	48	16	0.770	1.12 (0.45–2.76)	0.810
	Positive	27	6				59	31				12	45				13	5			

## Supplementary Materials

The following are available online at https://www.mdpi.com/2076-0817/10/1/83/s1, Table S1: Serological screening using three commercial ELISA, Table S2: Provirus detection in studied animals, Table S3: ELISA individual classification, Table S4: Animal classifications according to ELISA#1 and PCR results, Table S5: Animal classifications according to ELISA#2 and PCR results, Table S6: Animal classifications according to ELISA#3 and PCR results.
